# An effective DNA extraction protocol optimized for tropical swamp peat samples

**DOI:** 10.1128/spectrum.01373-25

**Published:** 2025-11-28

**Authors:** Júlia Brandão Gontijo, Gabriel Valverde Firmino, Jéssica Adriele Mandro, André Luiz Miranda Reis, Wanderlei Bieluczyk, Júlio César Feitosa Fernandes, Plínio Barbosa de Camargo, Jorge Luiz Mazza Rodrigues, Siu Mui Tsai, Pablo Vidal-Torrado

**Affiliations:** 1 University of São Paulo, Center for Nuclear Energy in Agriculture, Cell and Molecular Biology Laboratory, Piracicaba, São Paulo, Brazil; 2 Department of Land Air & Water Resources, University of California - Davis, Davis, California, USA; 3 Department of Soil Science, University of São Paulo, “Luiz de Queiroz” College of Agriculture, Piracicaba, São Paulo, Brazil; 4 University of São Paulo, Center for Nuclear Energy in Agriculture, Isotopic Ecology Laboratory, Piracicaba, São Paulo, Brazil; 5 Environmental Genomics and Systems Biology Division, Lawrence Berkeley National Laboratory, Berkeley, California, USA; Institute of Microbiology, Chinese Academy of Sciences, Beijing, China

**Keywords:** DNA extraction, microbial communities, 16S rRNA, *mcr*A, tropical peatlands

## Abstract

Peatlands are waterlogged ecosystems that store large amounts of carbon, harbor highly specialized endemic biodiversity, and play a crucial role in climate regulation. However, the microbial communities of tropical peatlands, particularly in the Atlantic Forest, remain largely unexplored. Molecular tools are essential for characterizing these communities, yet conventional DNA extraction kits are often ineffective for peat soils, likely due to their high organic matter content, low pH, and the presence of PCR inhibitors. This study aimed to optimize a conventional soil DNA extraction protocol to enhance DNA yield and purity from tropical peat soils, ensuring more reliable molecular analyses. The optimized protocol resulted in a four-times increase in DNA concentration compared to the conventional protocol and improved DNA purity. Agarose gel electrophoresis confirmed higher DNA integrity and also revealed the presence of RNA in samples subjected to the optimized protocol, indicating its potential for co-extraction of DNA and RNA. Quantitative PCR (qPCR) analysis showed significantly higher abundances of bacterial and archaeal 16S rRNA genes, as well as the *mcr*A gene, in optimized compared to conventional protocol, with statistically significant differences across soil profiles and depths. Furthermore, the optimized protocol yielded a lower coefficient of variation among replicates, enhancing reproducibility and consistency in microbial quantification. These findings demonstrate that the optimized protocol effectively extracts high-quality DNA from tropical peat soils, addressing common challenges associated with commercial kits. Its implementation can support improved future research on microbial-driven biogeochemical processes in tropical peatlands, particularly in the context of greenhouse gas emissions and climate change.

**IMPORTANCE:**

Tropical peatlands play a central role in carbon storage and greenhouse gas dynamics, yet their microbial communities remain largely unexplored due to analytical challenges. High organic matter content, low pH, and PCR inhibitors commonly limit the efficiency of molecular tools in these environments. This study presents an optimized DNA extraction protocol that improves yield, purity, and reproducibility across peat profiles. The protocol enhanced the detection of microbial marker genes, such as bacterial and archaeal 16S rRNA and *mcrA*, and showed potential for co-extraction of RNA, expanding possibilities for future multi-omic approaches. This methodological advance enables more accurate and consistent microbial analyses in tropical peat soils, contributing to a better understanding of microbial roles in biogeochemical cycles and climate-related processes.

## INTRODUCTION

Peatlands are wetland ecosystems that cover approximately 2.84% of the Earth’s surface and are composed primarily of peat, a unique soil layer formed from partially to highly decomposed organic matter. These ecosystems store vast carbon reserves, serving as critical carbon sinks by sequestering between 142 and 288 gigatons of carbon in thick, plant-derived layers (1, 2). For instance, carbon storage per area in tropical peatlands is exceptionally higher than in well-drained soils, averaging 2,000 tons per hectare for global estimates (3). Therefore, while these ecosystems play a pivotal role in global climate regulation (4, 5), their degradation could release massive amounts of greenhouse gases, particularly methane (CH_4_) and carbon dioxide (CO_2_), significantly contributing to global warming and climate change (2).

The formation of peat is driven by the continuous accumulation of plant detritus under conditions that markedly slow down decomposition. High water saturation and low oxygen concentrations inhibit most of the aerobic microbial activity, allowing the emergence of microbes adapted to anaerobiosis and favoring organic material to build up over time (4). Beyond their capacity for carbon sequestration, tropical swamp peatlands offer a range of essential ecosystem services, such as regulating hydrological cycles, maintaining water quality, and providing habitats for diverse endemic plant and animal species (1, 2). These multifaceted functions highlight the importance of studying and preserving these unique ecosystems, particularly given their role in mitigating climate change and supporting biodiversity.

A key feature of peatlands is their complex soil microbiota, which plays a fundamental role in driving biogeochemical cycles, especially the CH_4_ cycle (6). The shifting or permanent waterlogged conditions suppress aerobic microbes that generate CO_2_, while promoting anaerobic decomposition of the organic matter, particularly activating biogeochemical pathways of CH_4_ production (7, 8). Therefore, specific microorganisms, such as methanogenic archaea responsible for CH_4_ production, thrive in these environments (9). Considering that CH_4_ is the second most significant greenhouse gas, with a warming potential up to 28 times that of CO_2_ (10), understanding these microbial dynamics is crucial for assessing both ecosystem health and the broader impacts on climate.

Molecular tools, particularly quantitative PCR (qPCR), have become indispensable for investigating the above-mentioned microbial communities. Because the majority of soil microorganisms are difficult to culture, molecular markers are widely used to infer community structure and function. Markers, such as the *mcr*A gene, which encodes a subunit of the methyl-coenzyme M reductase enzyme found exclusively in methanogens (11), and the 16S rRNA gene, widely used for prokaryotic taxonomy (12), are essential for these analyses. To ensure reliable downstream analysis results, high-quality DNA is required, with purity commonly assessed through the 260/280 ratio, thereby minimizing the influence of PCR inhibitors (13).

Despite Brazil holding approximately 18%–20% of the world’s tropical peatlands, covering an estimated 611,000 hectares (14), molecular studies on their belowground microbial communities remain scarce. Many of these peatlands occur in the Atlantic Forest, a biome where natural vegetation cover is reduced to 36% of its original extent due to extensive degradation (15). The unique biogeochemical features of tropical peats, including a high content of humic substances, low pH, water saturation, and dense fibrous organic matter, challenge standard DNA extraction protocols, which were mostly designed for well-drained soils or aquatic environments with lower organic matter (16).

Given the ecological relevance of tropical peatlands and the importance of their microbiota as indicators of environmental change, we hypothesize that specific adjustments to commercial DNA extraction protocols can improve both the quantity and quality of DNA retrieved from these challenging substrates. To test this, we developed and applied tailored modifications to the DNeasy PowerLyzer Powersoil kit (Qiagen, Hilden, North Rhine-Westphalia, Germany) on peat samples collected from surface (20–30 cm) and deep (180–190 cm) layers in the Parque Estadual Campina do Encantado, Southeastern Brazil.

## MATERIALS AND METHODS

### Studied site

**Fig 1:**
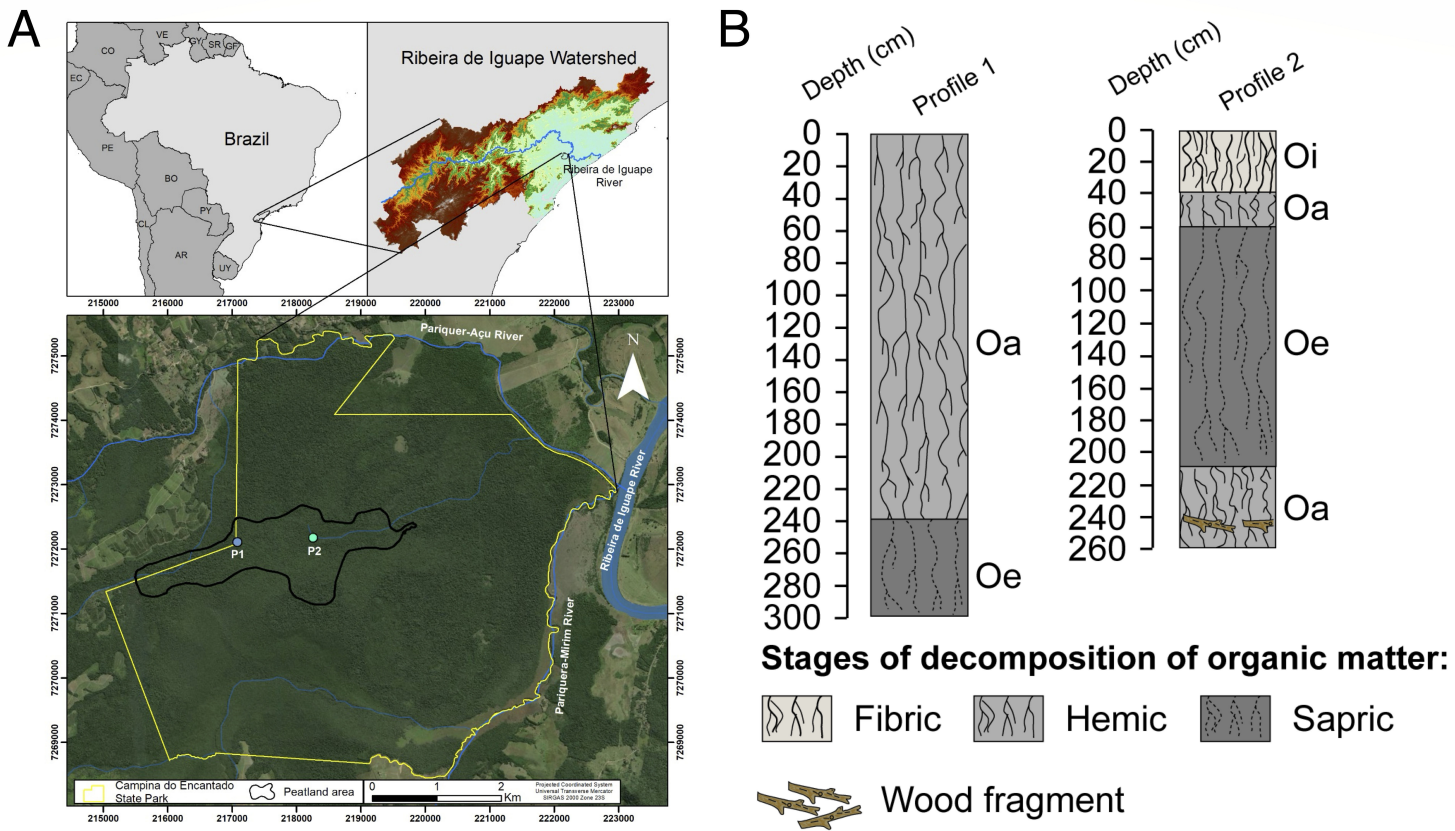
(**A**) Location of the study area within the Campina do Encantado State Park, located on the southern coast of São Paulo State, Brazil, indicating the locations of sampling sites P1 (24°38′17.90″ S, 47°47′22.93″ W) and P2 (24°38′17.87″ S, 47°46′56.99″ W). (**B**) Profiles of histosols, illustrating the different stages of organic matter decomposition. Horizon designations are based on Schoeneberger et al. (22) and Soil Survey Staff (23): Oa (sapric—highly decomposed), Oe (hemic—moderately decomposed), and Oi (fibric—slightly decomposed).

The Campina do Encantado State Park is located in the municipality of Pariquera-Açu, São Paulo, Brazil, covering an area of 3,000 hectares (Fig. 1A). Park headquarters lies west of the preservation area (24°38′52.48″ S, 47°48′44.72″ W; 16 m a.s.l.). According to the Köppen climate classification, the climate of the study area is Cfa (Humid Subtropical Climate, with hot summers and abundant, well-distributed rainfall throughout the year) (17). The area has altitudes below 10 m and is approximately 25 km from the coastline (18). The vegetation is characteristic of the Atlantic Forest biome, encompassing the Alluvial Dense Ombrophilous Forest (19–21), also known as swamp forest. This environment is dominated by tree species and features a high concentration of bromeliads in the lower stratum, close to the soil. Photos of the site are provided in Fig. S1 (supplemental material is found at https://doi.org/10.6084/m9.figshare.30535631).

### Peat sampling

We analyzed available satellite imagery and systematically cored the area using a peat corer to survey and verify peat thickness. Based on these previous steps, we selected two locations in a transect across the swamp forest, extracting two representative peat layers (20–30 and 180–190 cm), to be used in our laboratory experiment. Using a peat corer, two histosol profiles were sampled in an identified peat bog (see Fig. 1A). Profile 1 (P1) was sampled 1,000 m from the nearest forest edge at 24°38′17.90″ S, 47°47′22.93″ W, and Profile 2 (P2) at 1,700 m along the same transect at 24°38′17.87″ S, 47°46′56.99″ W. Both profiles were characterized following international guidelines for describing and classifying soil horizons (22, 23). This characterization is illustrated in Fig. 1B.

The sampled layers, 20–30 cm and 180–190 cm, were selected to represent a near-surface condition with predominantly fibric material and a deeper environment characterized by hemic/sapric features within the peat profile, thereby capturing contrasting stages of peat decomposition and organic matter composition. The average peat pH was approximately 3 at both sites and depths. The carbon content in layers 20–30 cm and 180–190 cm was 48.3% and 40.5% in P1, and 55.8% and 51.2% in P2, respectively. Immediately after sampling, the peat was transported to the laboratory under temperature conditions mimicking those of the field to preserve sample integrity. In the laboratory, the samples were promptly transferred to 2 mL microtubes in triplicate and stored at −80°C for subsequent analysis.

### Laboratory experiment design

 From each layer in both profiles, three samples were collected, adding up to a total of 12 samples. The samples were destined for DNA extraction using two different protocols, aiming to compare the efficiency of each of them for concentration and purity of DNA, followed by molecular analysis.

### DNA extraction and quantification

**Fig 2:**
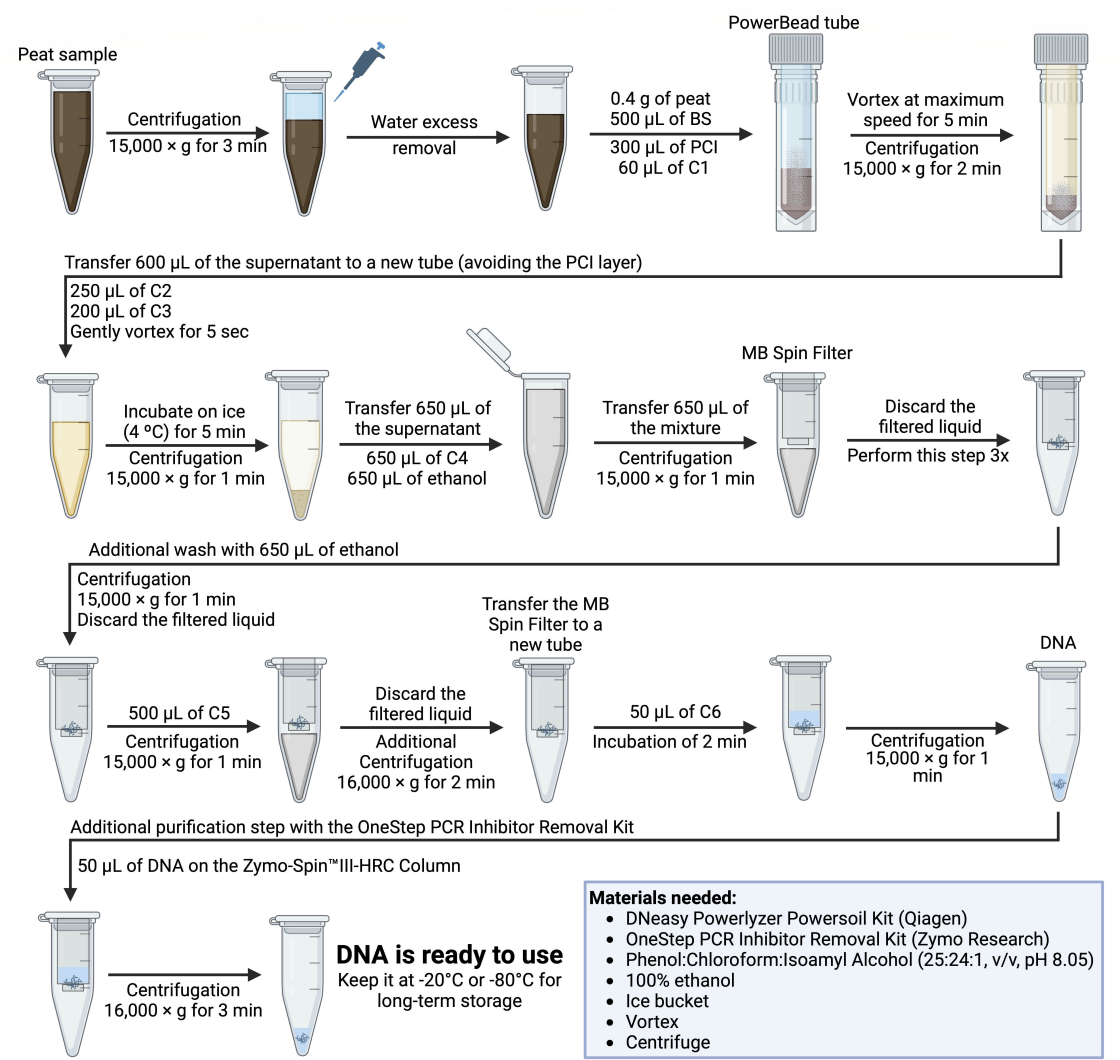
Step-by-step workflow of the optimized DNA extraction protocol for swamp peat samples. Figure created with BioRender.

We evaluated the efficiency of two DNA extraction protocols using the DNeasy PowerLyzer PowerSoil DNA Isolation Kit (Qiagen, Hilden, North Rhine-Westphalia, Germany). Initially, peat samples were processed using the manufacturer’s standard protocol (conventional protocol, CP). We then optimized the procedure (optimized protocol, OP), adapting an alternative protocol previously suggested by MoBio that incorporated phenol:chloroform:isoamyl alcohol in the lysis step and an additional ethanol wash, as described by Mantri et al. (24). Building on this framework, we introduced further modifications to improve DNA yield and purity. First, the 2 mL microtubes containing the peat samples were thawed and centrifuged at 15,000 × *g* for 3 min, and excess water was removed using a micropipette. To enhance DNA yield, the sample amount was increased from 0.25 to 0.4 g in the PowerBead tubes. Next, 500 µL of Power Bead Solution was added together with 300 µL of UltraPure phenol:chloroform:isoamyl alcohol (25:24:1, vol/vol), pH 8.05 (Invitrogen, Carlsbad, USA), and 60 µL of the C1 solution. The mixture was vortexed at maximum speed for 5 min and then centrifuged at 15,000 × *g* for 2 min. Subsequently, 600 µL of the supernatant was transferred to a new collection tube, and 250 µL of the C2 and 200 µL of the C3 solutions were added simultaneously; the mixture was vortexed for 5 s, incubated on ice for 5 min, and then centrifuged for 1 min at 15,000 × *g*. Thereafter, 650 µL of the resulting supernatant was transferred to a new tube and mixed with an equal volume of the C4 solution and 100% ethanol. During the DNA binding and cleaning steps using the MB Spin Filter, the samples were centrifuged for 1 min at 15,000 × *g*; this step was repeated three times. An additional cleaning step was performed by adding 650 µL of 100% ethanol, followed by centrifugation for 1 min at 15,000 × *g*. Then, 500 µL of the C5 solution was added and centrifuged under the same conditions. The MB Spin columns were subsequently centrifuged for 2 min at 16,000 × *g*. Finally, the MB Spin columns were transferred to new tubes, and the DNA was eluted in 50 µL of the C6 solution, allowing an incubation of 2 min to ensure efficient elution from the membrane, followed by a final centrifugation at 15,000 × *g* for 1 min. Upon completion of the extraction, an additional purification step was carried out using the OneStep PCR Inhibitor Removal Kit (Zymo Research, Murphy Ave, Irvine, California), following the manufacturer’s standard protocol. DNA must be kept at −20°C or −80°C for long-term storage. The complete, step-by-step, optimized protocol for extracting DNA from swamp peat samples has been provided in Fig. 2, and the full protocol is available in the Supplementary Information (supplemental material is found at https://doi.org/10.6084/m9.figshare.30535631). The total time required to complete the protocol for 12 samples is approximately 60 min.

Following DNA extraction with both protocols, we assessed DNA concentration and purity using three methods: electrophoresis on a 1% agarose gel, spectrophotometric analysis with a NanoDrop 2000c (260/280 ratio) (Thermo Fisher Scientific, Inc., Waltham, MA, USA), and fluorometric quantification with a Qubit Q32857 fluorometer using the dsDNA BR Assay Kit (Thermo Fisher Scientific, Inc., Waltham, MA, USA).

### qPCR of the 16S rRNA genes of Archaea and Bacteria and the *mcrA* gene

To compare the efficiency of the conventional (CP) and optimized (OP) protocols, real-time PCR (qPCR) was used to quantify the copy number of archaeal and bacterial 16S rRNA genes, using the primer pairs 519F/915R for Archaea, Eub338F/Eub518R for Bacteria, and mlas-F/mcrA-R for *mcr*A. The primers’ references, sequences, and amplification conditions are available in Table 1. For each gene, a standard curve was created, containing from 10 to 10^10^ copies of the gene of interest, obtained previously using the conventional PCR in environmental samples, using a Gene Amp PCR System 9700 thermal cycler (Thermo Fisher Scientific, Inc., Waltham, MA, USA). For both genes, the qPCR for each sample was performed in the StepOnePlus instrument (Thermo Fisher Scientific, Inc., Waltham, MA, USA), with a final volume of 10 µL, containing 5 µL of SYBR Green ROX qPCR Master Mix (Thermo Fisher Scientific, Inc., Waltham, MA, USA), 1 µL of each primer (5 pmol), 0.2 µL of bovine serum albumin (BSA) (20 mg mL^−1^) (Thermo Fisher Scientific, Inc., Waltham, MA, USA), 1.8 µL of DNAse and RNAse-free water, and 1 µL of DNA. The samples were standardized to 10 ng/µL, while those with lower concentrations were kept at their original values. These values were used in the gene abundance calculations. The results of the qPCR quantification were analyzed using StepOne Software v2.3 (Thermo Fisher Scientific, Inc., Waltham, MA, USA), later exported as spreadsheets, and converted to the number of copies per gram of soil.

**TABLE 1:** Set of primers and references for each gene used in this study

Gene	Primer	Sequence (5′-3′)	Reference	PCR conditions
16S rRNA *Archaea*	519F	CAGCCGCCGCGGTAA	(25)	95°C for 10 min; 40 cycles: 95°C for 30 s, 53°C for 40 s, 72°C for 40 s; melting curve: 95°C for 10 s, 53°C for 1 min, 95°C for 15 s
	915R	GCCATGCACCWCCTCT	(26)	
16S rRNA *Bacteria*	Eub338F	GCTGCCTCCCGTAGGAGT	(27)	95°C for 10 min; 40 cycles: 94°C for 15 s, 56°C for 30 s and 72°C for 45 s; melting curve: 95°C for 15 s, 56°C for 1 min, 95°C for 15 s
	Eub518R	ATTACCGCGGCTGCTGG		
*mcr*A	mlas-F	GGYGGTGTMGGDTTCACMCARTA	(28)	95°C for 10 min; 45 cycles: 95°C for 30 s, 60°C for 45 s and 72°C for 30 s; melting curve: 95°C for 15 s, 60°C for 1 min and 95°C for 15 s
	mcrA-R	CGTTCATBGCGTAGTTVGGRTAGTT	(29)	

### Data analysis

Statistical analyses and graphical visualizations were performed using RStudio 4.2.2. The results include DNA quantification using NanoDrop and Qubit, as well as qPCR analysis of 16S rRNA genes for total archaeal and bacterial communities and *mcr*A for methanogens. Data normality was assessed using the Shapiro-Wilk test, and the Kruskal-Wallis test was applied to compare protocols within each Depth and Site, with Bonferroni correction for multiple comparisons. All statistical analyses were conducted using the ggpubr package (version 0.6.0 [30]), while plots were generated with ggplot2 (version 3.0.0 [31]).

## RESULTS AND DISCUSSION

Tropical peatlands play a crucial role in global biogeochemical cycles, particularly C and CH₄ dynamics (32). However, their microbial communities remain poorly understood, preventing our ability to fully comprehend their ecological functions and responses to environmental changes. Environmental DNA extraction from tropical peatlands presents significant challenges due to their high organic matter content, low pH, and the presence of PCR inhibitors, such as complex organic compounds commonly referred to as humic substances (16). Although commercial kits are designed for soil samples, they are not specifically adapted to the complex composition of tropical peat. These limitations often result in low DNA yield and compromised purity, underscoring the need for optimized extraction protocols tailored to these challenging conditions.

**Fig 3:**
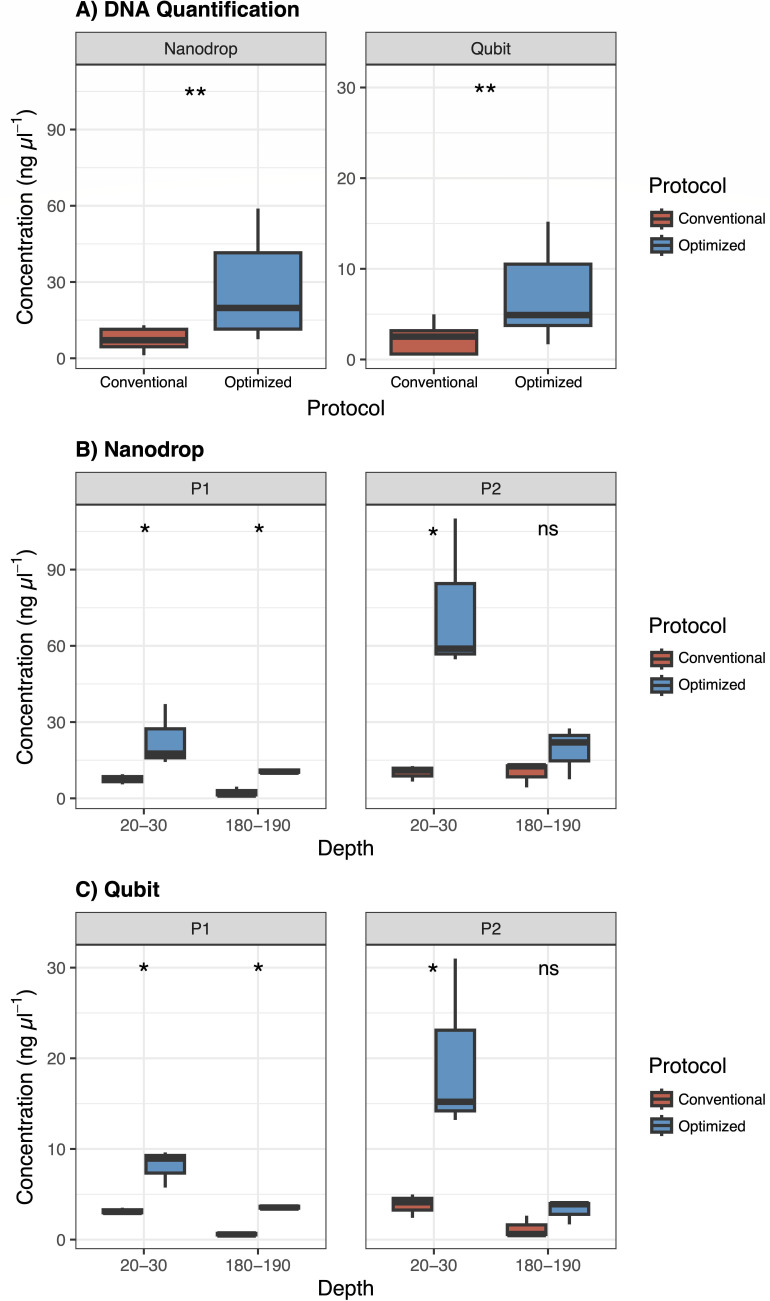
(**A**) DNA quantification comparing the conventional (CP) and optimized (OP) protocols, measured using NanoDrop and Qubit across all samples. (**B**) NanoDrop and (**C**) Qubit quantifications considering the effects of the CP and OP on DNA yield, with comparisons across different profiles (P1 and P2) and depths (20–30 cm and 180–190 cm). Asterisks indicate statistically significant differences between treatments, determined by Kruskal-Wallis tests with Bonferroni correction (*P < 0.05, **P < 0.01, ns = not significant).

In the face of these demanding conditions, an optimized DNA extraction protocol (OP) was developed, resulting in a four times increase in DNA concentration compared to the conventional protocol (CP), as quantified by NanoDrop and Qubit measurements (Fig. 3; Table 2). On average, across all treatments, the total DNA yield increased from 750.8 to 1,591.7 ng (NanoDrop) and from 221.2 to 433.75 ng (Qubit). This improvement is attributed to key adaptations, including the initial peat sample weight (from 0.25 to 0.4 g) that increased the total amount of DNA being recovered (33), and the use of a phenol-chloroform-isoamyl alcohol solution, which enhanced protein solubilization and the removal of organic contaminants (34). Additionally, the sequential incorporation of solutions C2 and C3, both targeting similar organic and inorganic inhibitors, combined with an incubation step on ice, enhanced the precipitation of contaminants, thereby improving DNA purity and saving processing time. Furthermore, the additional ethanol wash contributed to improved DNA precipitation and more effective removal of impurities, as it prevents the dissolution of DNA that remains bound to the membrane while other sample components are washed away (35).

**TABLE 2:** Mean and standard deviation of Qubit and NanoDrop concentrations (ng µL^−1^), and 260/280 ratio values

Profile	Depth (cm)	Protocol	Qubit (ng µL^−1^)	NanoDrop (ng µL^−1^)	260/280
P1	20–30	Conventional	3.14 ± 0.34	7.53 ± 2.00	1.52 ± 0.18
		Optimized	8.1 ± 2.07	23 ± 12.32	1.60 ± 0.20
	180–190	Conventional	0.62 ± 0.18	2.43 ± 1.88	2.48 ± 0.78
		Optimized	3.59 ± 0.21	10.77 ± 0.93	1.82 ± 0.15
P2	20–30	Conventional	3.83 ± 1.31	10.1 ± 3.15	1.52 ± 0.20
		Optimized	19.8 ± 9.75	74.57 ± 30.84	1.51 ± 0.18
	180–190	Conventional	1.25 ± 1.20	9.97 ± 4.91	1.44 ± 0.02
		Optimized	3.21 ± 1.33	19 ± 10.33	1.57 ± 0.08

A final purification step using the OneStep PCR Inhibitor Removal Kit (Zymo Research, Murphy Ave, Irvine, California) was included to increase DNA purity. Accordingly, the 260/280 ratio obtained from NanoDrop, the optimal range of 1.8 ± 0.2, is expected to be an indicator of DNA purity (36). Although challenging, the OP improved DNA quality by increasing the number of samples within this range (Table 2). Additionally, agarose gel electrophoresis confirmed the higher yield and high quality of the extracted DNA, while also indicating the presence of RNA in OP samples (Fig. S2 [supplemental material is found at https://doi.org/10.6084/m9.figshare.30535631]). Although RNA extraction was not the primary objective, its co-extraction suggests that this protocol may be useful for future studies investigating active microbial communities in tropical peatlands. The co-extraction of DNA and RNA allows for a more comprehensive analysis of microbial composition and activity, crucial for understanding ecosystem dynamics (37). However, additional precautions should be taken to ensure RNA integrity for such applications, such as proper sample preservation for RNA integrity, strict RNase-free handling, immediate stabilization of RNA, and the use of DNase treatment to separate RNA from DNA (38).

**Fig 4:**
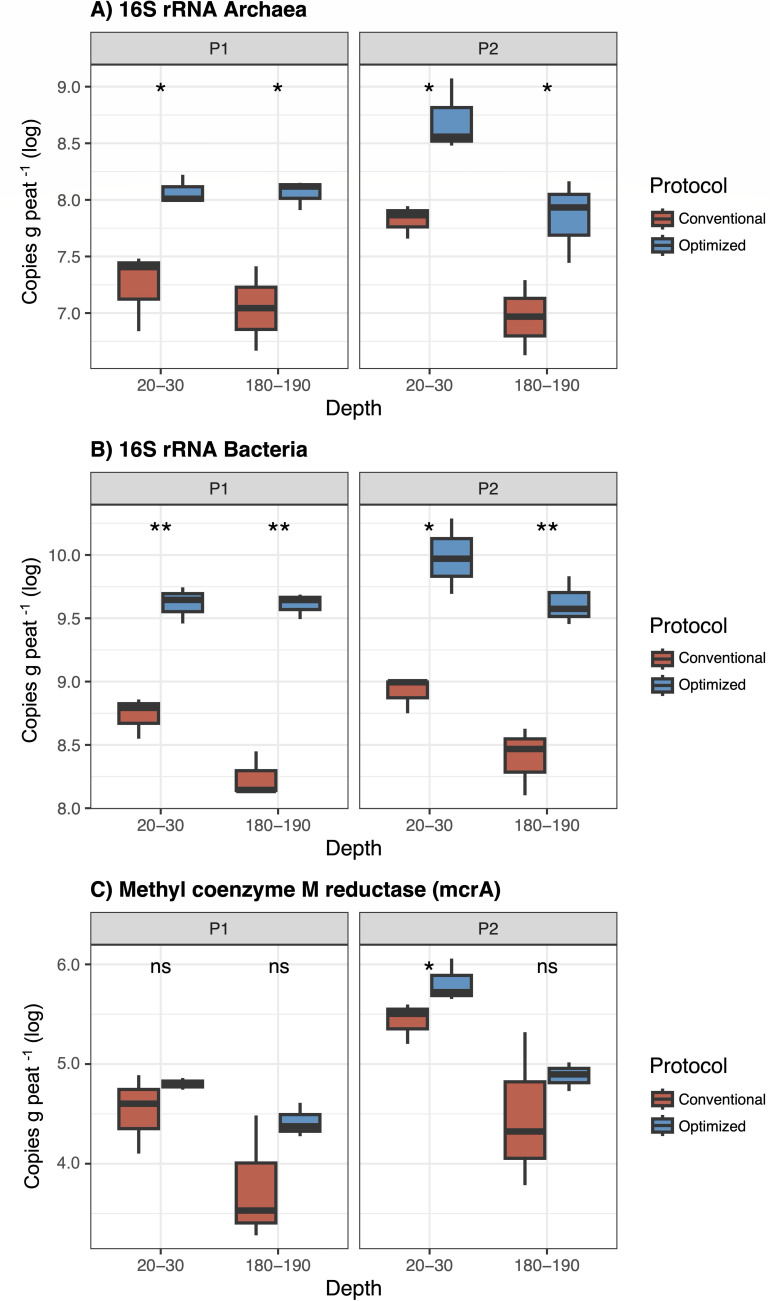
qPCR analysis of (**A**) archaeal 16S rRNA, (**B**) bacterial 16S rRNA, and (**C**) *mcr*A genes, evaluating the effects of the conventional (CP) and optimized (OP) protocols on the gene’s quantification. Comparisons were performed across different profiles (P1 and P2) and depths (20–30 cm and 180–190 cm). Data are presented on a logarithmic scale (log). Asterisks indicate statistically significant differences between treatments, determined by Kruskal-Wallis tests with Bonferroni correction (*P < 0.05, **P < 0.01, ns = not significant).

Since the choice of DNA extraction method directly influences the purity and yield of nucleic acids, which in turn affects the accuracy of downstream molecular analyses (39), we evaluated the suitability of DNA extracted using the conventional and optimized protocols for qPCR-based quantifications. qPCR assays targeted bacterial and archaeal 16S rRNA genes. Furthermore, the *mcr*A gene was included in the analysis due to the high CH_4_ emissions characteristic of the study profiles, especially in P2 (Table S1 [supplemental material is found at https://doi.org/10.6084/m9.figshare.30535631]), which suggests a high abundance of methanogenic archaea in the samples. To mitigate potential PCR inhibition, BSA was added to the qPCR reactions. BSA is a transport protein that can interact with lipids and organic molecules, effectively minimizing different types of PCR inhibitors (40) and enhancing amplification efficiency in samples with inhibitory compounds (41). The assays exhibited high linearity (R^2^ > 0.94) with efficiencies ranging from 92% to 101%, confirming the reliability of the results. Figure 4 presents the quantification of the studied genes on a logarithmic scale, expressed as copies per gram of peat, while Table S2 (supplemental material is found at https://doi.org/10.6084/m9.figshare.30535631) provides the average of the gene quantification per treatment.

The abundances of archaeal and bacterial 16S rRNA genes were significantly higher in OP (averaging 2.3 × 10^8^ and 6.0 × 10^9^ copies per g peat^−1^, for Archaea and Bacteria, respectively) compared to CP (2.9 × 10^7^ and 4.7 × 10^8^ copies per g peat^−1^, respectively) (Fig. 4A and B), with statistically significant differences across both profiles and depths (*P* < 0.05). Although *mcr*A gene abundance tended to be higher in OP (1.1 × 10^5^ in CP versus 2.2 × 10^5^ in OP), statistical significance was only observed at the 20–30 cm depth in P2 (Fig. 4C). Furthermore, qPCR results from OP exhibited a lower variation coefficient for the three studied genes among samples compared to CP (0.041 in CP versus 0.034 in OP for 16S rRNA *Archaea*, 0.022 in CP versus 0.019 in OP for 16S rRNA *Bacteria*, and 0.117 in CP versus 0.029 in OP for *mcr*A). Therefore, in addition to increasing DNA yield and quality, OP also enhanced reproducibility in downstream DNA analyses. This stability is particularly important for comparing microbial communities across treatments and environmental conditions, as it strengthens the reliability of observed trends and ecological interpretations (33). Importantly, Thermo Scientific (36) emphasizes that the most reliable indicator of DNA extraction quality is its performance in downstream applications. In this sense, the high-quality DNA extracted through OP is well-suited for metagenomic studies, enabling a broader exploration of microbial diversity and functional potential. Future metagenomic research using this optimized DNA extraction method can further investigate the role of microbial communities in biogeochemical cycles, particularly in relation to climate change.

### Concluding remarks

The efficiency of the optimized protocol demonstrates its suitability for extracting high-quality DNA from tropical peat soils, overcoming common limitations associated with commercial extraction kits. While qPCR was used in this study to validate DNA quality, the extracted DNA is also suitable for high-throughput applications, such as metagenomics, expanding its potential for microbial community profiling. Additionally, the protocol showed promise for simultaneous DNA and RNA extraction, which is particularly valuable for studying both taxonomic composition and gene expression in microbial communities; however, further optimization of the co-extraction process will be necessary in future applications. The ability to obtain high-quality nucleic acids from tropical peatlands is crucial for advancing reliable research on microbial-driven biogeochemical processes, particularly in the context of climate change. Future investigations utilizing this optimized protocol can further explore the role of peatland microbiomes in ecosystem services, contributing to a better understanding of their ecological resilience and response to environmental disturbances.
